# Fixating on a laser-projected point at a constant distance suppresses periodic eye movements and impairs balance control during cycling

**DOI:** 10.1007/s00221-025-07133-5

**Published:** 2025-07-30

**Authors:** Takashi Kojima, Katsuki Cho, Masahiro Kokubu

**Affiliations:** 1 Graduate School of Comprehensive Human Sciences, University of Tsukuba, 1-1-1 Tennodai, Tsukuba, 305-8574 Ibaraki Japan; 2 Institute of Health and Sport Sciences, University of Tsukuba, 1-1-1 Tennodai, Tsukuba 305-8574 Ibaraki, Japan

**Keywords:** Cycling, Eye movement, Optokinetic nystagmus, Fixation, Texture

## Abstract

The visual field is influenced by movements of the body, head, and eye movements during self-motion. Therefore, compensatory eye movements that stabilize the visual field are crucial for perceptual-motor control. In cycling, a type of eye movement known as optokinetic nystagmus may help prevent visual blur and support the use of retinal flow for maintaining dynamic balance. This study aimed to examine the impact of visual environmental manipulation on dynamic balance control in cycling by altering gaze behavior. Participants cycled under three conditions and were instructed to ride as slowly as possible while staying on the path. In the control condition, they cycled along a striped path. In the laser condition, they cycled on the same path while fixating on a laser point projected 4 m ahead of the bicycle. In the monochrome condition, participants cycled along a plain white path. Each condition consisted of five trials. The results showed that the amplitude at the peak frequency of eye movements was significantly lower in the laser condition compared to the control and monochrome conditions. In addition, the cycling distance and duration were significantly shorter in the laser condition compared to the control condition. There were no significant differences in eye movements or cycling performance between the control and monochrome conditions. These results suggest that fixating on a laser point projected at a constant distance ahead suppresses the amplitude of eye movements and impairs balance control during cycling. Natural gaze behavior is likely to be an important factor for cycling safely.

## Introduction

Since the proposal of the link between perception and action (Gibson 1958), the role of visual information in locomotor behaviors such as steering (Lappe et al. 1999; Warren 1998), collision avoidance (Lee 1976; Yilmaz and Warren 1995; Fajen 2007), and interception (Chapman 1968; Fink et al. 2009) has been extensively studied. Early research employed methods that manipulated simulated visual environments on a screen to measure judgments of heading direction and the braking control. This approach led to the development of mathematical models and empirical experiments examining the relationship between task-specific optical variables and movement control (Warren 1988). However, body, head, and eye movements influence the optic flow of the environment during real-world self-motion, resulting in complex retinal flow. In recent years, advancements in measurement devices and image processing technologies have made it possible to accurately measure retinal flow during locomotion in natural environments by using full-body inertial sensors and eye-tracking equipment (Matthis et al. 2018). As a result, it has been proposed that retinal flow may contribute to the dynamic balance control during walking (Matthis et al. 2022). Since the contribution of this visual kinesthesis to motor control is considered to be independent of the mode of locomotion (Gibson 1979), it is important to investigate whether similar mechanisms apply in other contexts as well. In this regard, cycling presents a particularly relevant task. In addition to walking, cycling is a form of locomotion that also demands dynamic balance. Unlike walking, where the vestibulo-ocular reflex (VOR) stabilizes gaze during head motion (Matthis et al. 2022), cycling typically involves a more stable head posture, allowing for the observation of optokinetic nystagmus (OKN)—a compensatory eye movement in response to large-field motion. These mechanisms suggest that retinal flow stabilized by eye movements could support balance control. However, there is currently limited evidence supporting the contribution of retinal flow to the dynamic balance control of cycling. Therefore, this study investigated whether retinal flow contributes to cyclists’ dynamic balance control. This research could provide valuable insights for situations that demand particularly high balance ability, such as riding on uneven terrain or alongside busy roads. Furthermore, this research contributes to the broader understanding of how humans employ retinal flow information derived from eye movements to regulate dynamic balance.

A state of maintaining dynamic balance refers to the state to ride a bicycle with both front and rear wheels without falling. For a bicycle to be ridden without falling, the combined center of mass of the cyclist and the bicycle must remain within the base of support defined by the front and rear wheels. To maintain dynamic balance, cyclists must either shift the center of mass by tilting the body or the bicycle, or adjust the base of support by steering the handlebars. This movement is referred to as the cyclists’ dynamic balance control. As such, riding on narrow paths, where steering is limited, poses a challenge to maintaining dynamic balance and requires a higher level of balance control from cyclists. Indeed, cyclists with higher balance control, such as those who consistently ride more than 3,000 km annually for over five years, exhibit smaller deviations from the centerline compared to cyclists with lower balance control, who ride fewer than 100 km annually (Fonda et al. 2017). Additionally, competitive race participants demonstrate less body lean compared to daily commuters (Cain et al. 2016). Moreover, the ability of cyclists to maintain dynamic balance is particularly required in cycling at low-speed. It is known that bicycles can remain upright and self-stabilize within a certain speed range even without the operation of cyclists (Meijaard et al. 2007), due to factors such as the gyroscopic effect of the wheels (Klein and Sommerfeld 1910), the positive trail of the front wheel (Jones 1970), and the mass distribution of the front wheel (Kooijman et al. 2011). In other words, reduced self-stability at low speeds demands compensatory balance control from the cyclist. Therefore, in this study, we assessed cyclists’ balance control by evaluating their ability to maintain stability while cycling at low speeds without deviating from a straight and narrow path.

Cycling on straight path, which does not involve directional control, is also well-suited for examining the influence of visual information on dynamic balance. A study analyzing gaze allocation during riding tasks with varying path widths and speeds reported that, when riding at lower speeds on narrower paths, a greater proportion of gaze was directed toward the path itself (Vansteenkiste et al. 2013). Additionally, it has been reported that cyclists spend more time fixating on the path when riding on narrow, unpaved paths compared to riding on maintained roads in real-world cycling scenarios (Vansteenkiste et al. 2014). This finding suggests that during cycling on a narrow path at low speeds, where maintaining dynamic balance is particularly challenging, visual information from the path may provide valuable cues for cyclists’ control. Furthermore, it has been observed that OKN, a repetitive periodic eye movement alternating between distant and near targets, occurs under such conditions (Vansteenkiste et al. 2013; Kojima and Kokubu 2024). OKN is characterized by a repetitive eye movement consisting of a slow phase that the eyes follow visual image, and a fast phase where the eyes quickly return to the initial position. The slow phase corresponds to the optokinetic response (OKR), which serves to stabilize the retinal image relative to the visual environment (Lappi 2016). In walking, it is well established that the vestibulo-ocular reflex VOR stabilizes the visual field by compensating for head movements, and it has been suggested that such compensatory eye movements during locomotion contribute to the generation of retinal flow useful for movement control (Matthis et al. 2022). In other words, in the absence of compensatory eye movements, the velocity vector of retinal flow could become larger, potentially impairing perceptual-motor control. To investigate this possibility, the present study aimed to examine the effects of suppressing eye movements using two different methods on cycling stability.

One visual environment involved the use of a fixation point, which was intended to manipulate the gaze direction and influence cycling movements. In experiments using visual stimuli, it has been reported that introducing a fixation point suppress OKN (Wyatt and Pola 1984; Pola et al. 1992). Furthermore, fixation on an LED attached to an eye mask has been shown to reduce the peak velocity of the slow phase of spontaneous nystagmus in a static standing posture (Jahn et al. 2002). In this study, we projected a laser point from a bicycle onto the ground at a fixed distance ahead of the cyclist. This point moved consistently with the bicycle, and participants were instructed to maintain their gaze on this fixation point.

Another visual environment was characterized by a reduction in ground texture. Previous studies have demonstrated that OKN is primarily induced by optic flow generated from ground texture (Lappe et al. 1998; Lappe and Hoffmann 2000; Niemann et al. 1999). Given that human OKN is predominantly driven by visual stimuli within the central fovea, it is plausible that the texture of the riding path influences OKN behavior during cycling. Accordingly, in the present study, a striped path was used for the control condition, whereas a monochromatic path with reduced texture was employed for the experimental condition.

In summary, this study examined whether two types of visual environments that manipulate gaze could suppress eye movements and impair cyclists’ dynamic balance control. The first visual environment investigated the effect of fixating on a laser point projected at a constant distance ahead of the cyclist while moving. The second visual environment examined the effect of reducing texture on the cycling path. We assume that these investigations will provide insight into how visual environments that suppress eye movements affect dynamic balance during cycling.

## Methods

### Participants

The participants consisted of 18 university students (23.3 ± 2.0 y, 168.9 ± 6.5 cm, 61.2 ± 12.5 kg, 11 males and seven females). An a priori power analysis (G*Power 3.1) for a repeated measures analysis of variance indicated a minimum sample size of 18 participants (β = 0.95, α = 0.05, effect size f = 0.40). The effect size was determined based on previous studies that observed the suppression of OKN by visual fixation (Wyatt and Pola 1984) and the effects of OKN suppression on postural sway (Jahn et al. 2002). All participants had prior experience riding bicycles; however, none had experience in cycling competitions such as road racing. All the participants provided written informed consent before participating in the study. This study was approved by the local ethics committee. All experiments were conducted according to the principles of the Declaration of Helsinki.

### Instrumentation

#### Bicycle

**Fig. 1:**
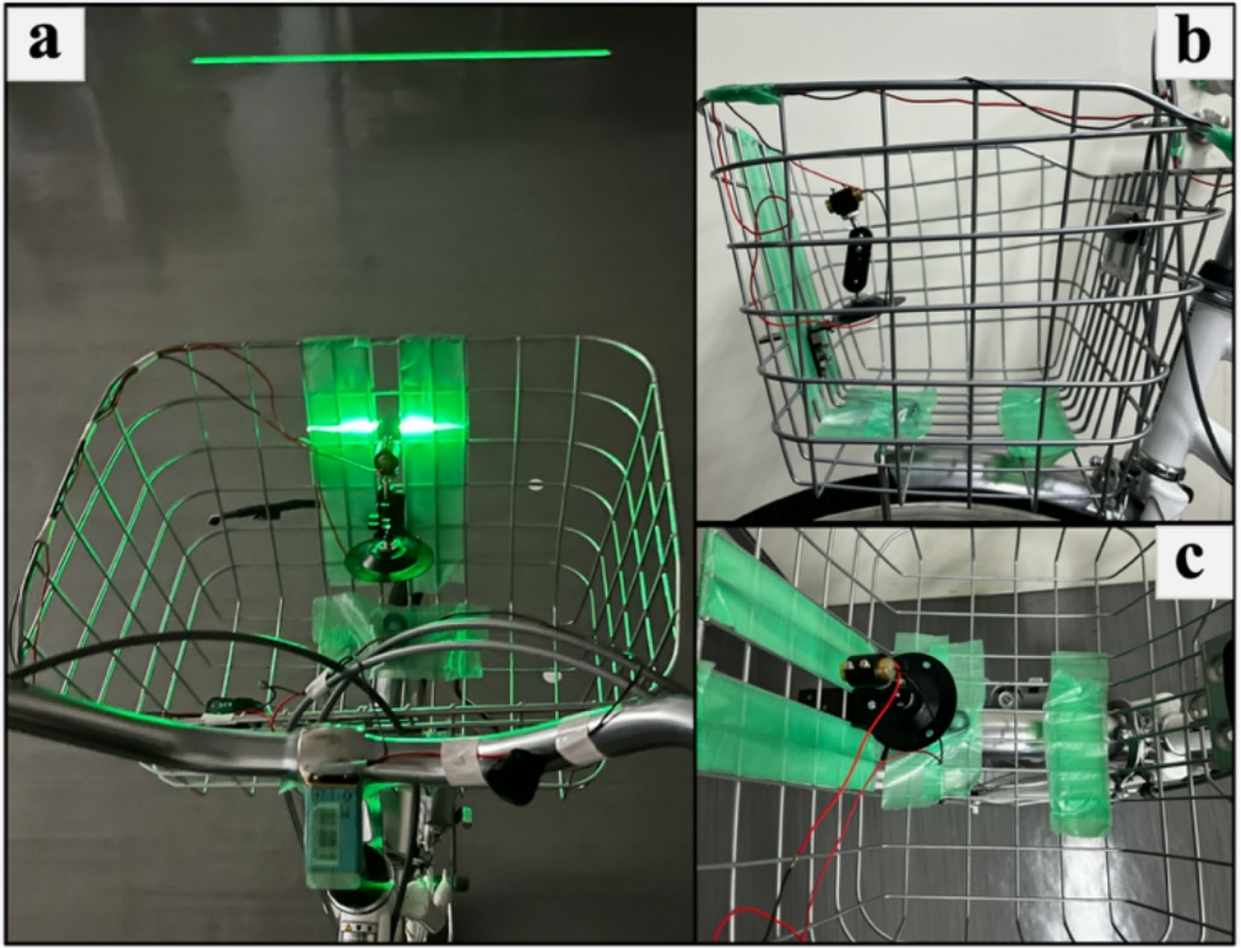
Setup of a laser point: **a** The laser projects a horizontal line 4 m ahead of the bicycle. **b** Top-down view of the attachment. **c** Side view of the attachment

All participants performed the cycling trial on a commuting bicycle (CASURY RELAX-L, ASAHI Co., Ltd., Japan) equipped with a six-speed manual transmission and 27-inch wheels. The handlebar was of the semi-up type. The gear ratio was restricted to 36 × 18, and the tire pressure was set to 400 kPa. A laser device (wavelength: 510–530 nm, output power: 0.39 mW, model: VLM-520–28 LPT, Quarton, Inc., Taiwan) was attached to the bicycle, projecting a laser point onto the path, which served as the fixation point (Fig. 1).

#### Paths

**Fig. 2:**
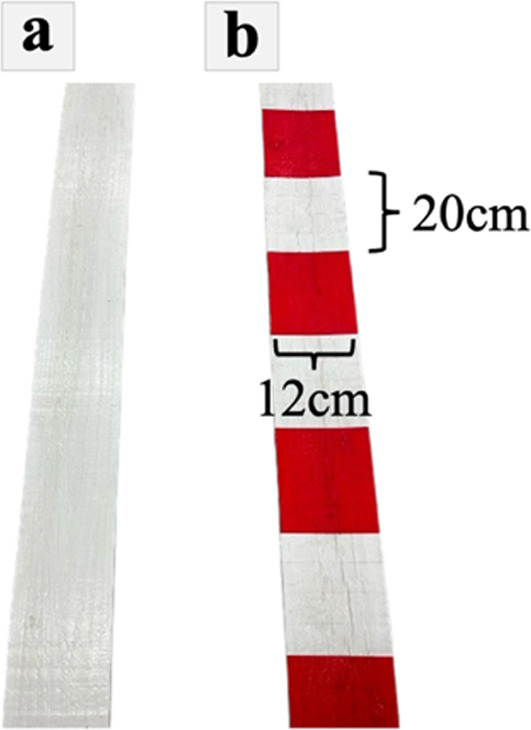
**a** White board was used in the monochrome condition. **b** Stripe board was used in the control and laser conditions

The path was composed of a wooden board, 20 m in length, 12 cm in width, and 13 mm in height. The monochrome path was made by affixing white plastic tape along the surface (Fig. 2a), whereas the stripe path was made by alternating white and red plastic tapes. The width of the stripes was 20 cm (Fig. 2b).

#### Measuring devices

The participants wore a mobile eye-tracking device (EMR-9, Nac Image Technology Co., Ltd., Japan) to record eye movements. The system detected the position of both pupils, recorded eye movements at a sampling frequency of 240 Hz, and captured videos of the experimental scenes using a 30 Hz video camera. The point of fall from the path was confirmed using a video camera. An inertial measurement unit (IMU) was mounted on the top of the helmet, and tilt angles were recorded at a sampling rate of 200 Hz.

### Task

The participants rode commuting bicycles. They were instructed to ride on the path as slowly as possible without falling off. When the participant fell off the path, cycling was terminated and the next trial was conducted.

### Conditions

The participants performed the task under three conditions. In the control condition, they rode on a board with a striped pattern. In the laser condition, they were instructed to ride on the striped board and fixate on a laser point projected 4 m ahead of the bicycle. The fixation distance was determined based on a previous study which measured gaze direction while cycling on a straight and narrow path (Kojima and Kokubu 2024). In the monochrome condition, the participants rode on a white board with no visual texture.

### Procedure

The saddle height for each participant was individually adjusted using the heel-to-pedal method, in which the saddle height was set to align with the bottom of the pedal stroke when the knee was fully extended with the heel on the pedal (Peveler et al. 2005; Fonda et al. 2017). Participants first completed five practice trials under each condition to familiarize themselves with the task without inducing fatigue. After a short rest period, they then performed five measurement trials for each of the three conditions while wearing the eye-movement recording devices. The order of conditions was counterbalanced across the participants.

### Parameters

Eye movements were recorded while cycling on the path using an eye-movement measuring device. Eye movements were analyzed by performing a discrete Fourier transform (DFT) using the “fft” function in MATLAB (MathWorks, USA) (see Eqs. 1and 2), followed by calculating the peak frequency and the amplitude using the single-sided amplitude spectrum (see Eqs. 3 and 4). OKRs are known to persist for less than 1 s (Authié et al. 2011; Balaban et al. 2017). Therefore, the highest spectral peak above 1 Hz was selected as the peak frequency of periodic eye movements, and the corresponding vertical-axis value at that peak frequency was considered the amplitude. First, the DFT was performed (see Eq. 1), followed by the calculation of the double-sided amplitude spectrum (see Eq. 3), and subsequently the single-sided amplitude spectrum was derived (see Eq. 4). In these formulations, *X(j)* denotes the *j*_th_ value of the time series data, *Y(k)* represents the *k*_th_ frequency component, *W*_*n*_ refers to the rotational vector of the complex exponential function on the unit circle, *i* is the imaginary unit, and *n* indicates the number of data points.

1 $$ \begin{array}{*{20}l} {Y\left( k \right) = \mathop \sum \limits_{{j = 1}}^{n} X\left( j \right) \cdot W_{n} ^{{\left( {j - 1} \right)\left( {k - 1} \right)}} } \\ \end{array} $$

2 $$\begin{array}{*{20}{l}} {~{W_{n~}}=~{e^{\frac{{ - 2\pi i}}{n}}}~} \end{array}$$

3 $$\begin{array}{*{20}{l}} {~P\left( k \right)=~\left| {\frac{{Y\left( k \right)}}{L}} \right|,~~~k=1,~2,~ \ldots,~\frac{L}{2}~+1,} \end{array}$$

4 $$\begin{array}{*{20}{l}} {~P^{\prime}\left( k \right)=~\left\{ {\begin{array}{*{20}{c}} {P\left( k \right)}&{~{\text{if}}~k=1~~{\text{or}}~~k=~\frac{L}{2}+1,} \\ {2 \cdot ~P\left( k \right)}&{{\text{otherwise}}{\text{.}}} \end{array}} \right.~} \end{array}$$

where *P(k)* denotes double-sided amplitude spectrum, *L* denotes the length of signal, and *P’(k)* denotes single-sided amplitude spectrum.

The peak frequency and its corresponding amplitude were used as indicators of eye movement. The peak frequency reflects the vertical oscillatory component of eye movements relative to the head. The amplitude at peak frequency represents the magnitude of the most dominant eye movement. The standard deviation (SD) of head tilt angle was calculated for each trial. The cycling distance and duration were measured as indices of cycling performance. Cycling distance was defined as the distance that the participant traveled before falling from the lane. Cycling duration was defined as the time for which they stayed in the lane. Cycling speed was calculated by dividing the cycling distance by cycling duration.

### Analysis

Three participants were excluded from analysis. Two were excluded because they did not direct their gaze toward the path in either the control or the monochrome condition. One participant was excluded because he did not fixate on the laser point as instructed in the laser condition.

The amplitude at the peak frequency, peak frequency, cycling distance, and cycling duration were compared across conditions using one-way repeated measures ANOVA. The Mauchly’s test indicated a violation of the sphericity assumption for cycling distance and head tilt angle; therefore, the degrees of freedom were adjusted using the Greenhouse-Geisser correction for statistical analysis. Multiple comparisons with Bonferroni correction were performed as post hoc tests. All statistical tests were performed using IBM SPSS software version 27 (SPSS Inc., USA), and statistical significance was set at *p* <.05.

## Results

**Fig. 3:**
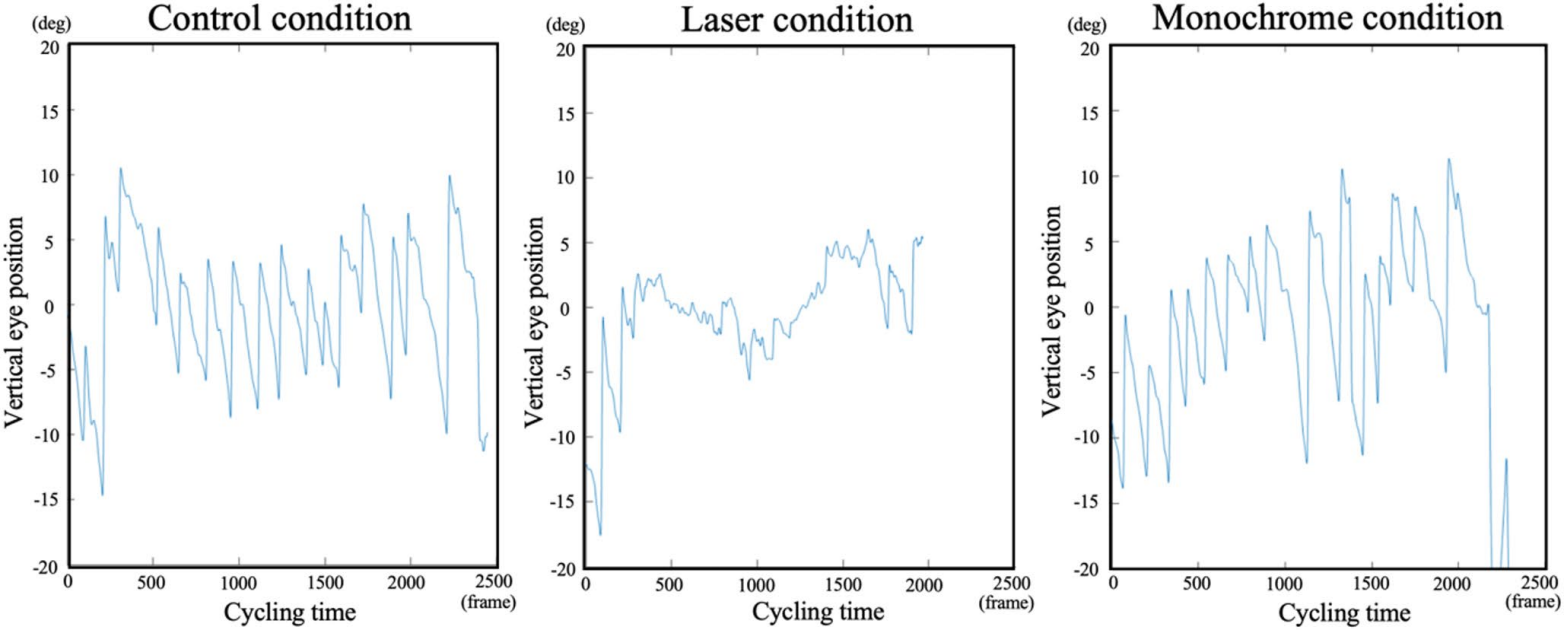
Eye movement data during one trial for a representative participant. (Left) Control condition, (Middle) Laser condition, (Right) Monochrome condition

**Fig. 4:**
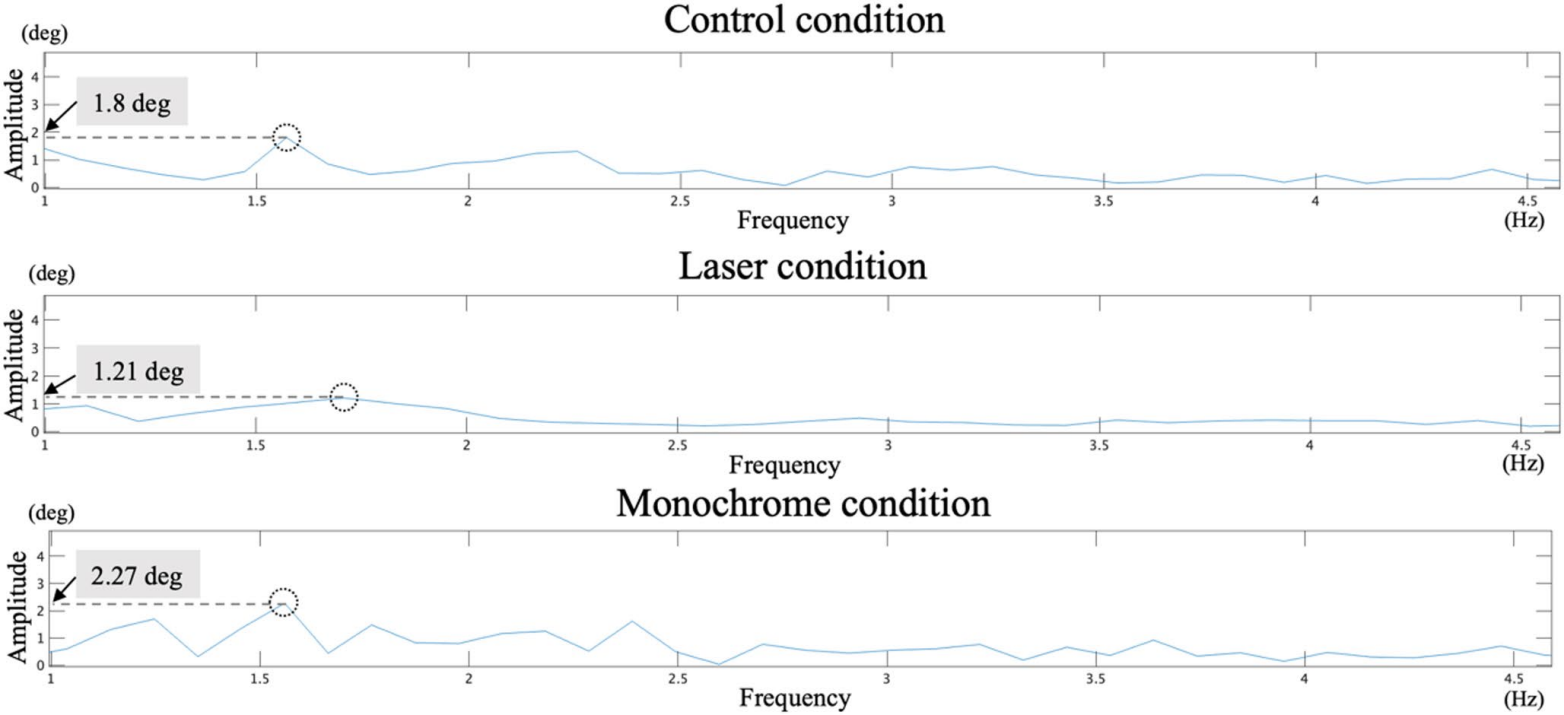
Graph of the frequency analysis of eye movements for a representative participant. (Upper) Control condition, (Middle) Laser condition, (Lower) Monochrome condition. The circles represent the detected peak frequencies, with the number indicating the amplitude at the peak frequency

**Fig. 5:**
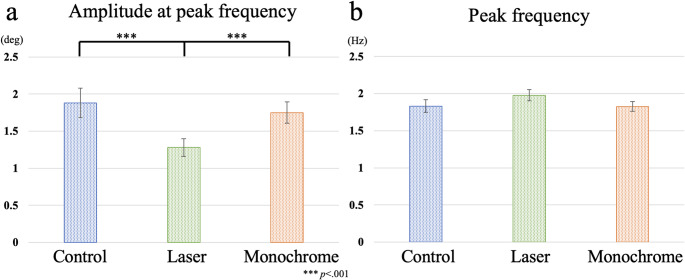
**a** Amplitude at peak frequency and **b** peak frequency in the control, laser and monochrome conditions. The error bars represent the standard error (SE)

Typical examples of eye movement and frequency analysis results for each condition are shown in Figs. 3 and 4. A one-way ANOVA revealed a significant main effect of condition on the amplitude at the peak frequency of eye movements (*F*(2, 28) = 18.641, *p* <.001, *η*_*p*_^*2*^ = 0.571; Fig. 5a). Post-hoc multiple comparisons with Bonferroni correction showed that the amplitude at peak frequency in the laser condition was significantly lower than that of the control (*p* <.001) and monochrome conditions (*p* <.001) (Control: 1.88 ± 0.76 Hz, Laser: 1.28 ± 0.47 Hz, Monochrome: 1.75 ± 0.56 Hz). The results indicated that eye movement amplitude was suppressed in the laser condition. In contrast, the peak frequency of eye movements was not significantly different among conditions (*F*(2, 28) = 1.992, *p* =.155, *η*_*p*_^*2*^ = 0.125) (Control: 1.83 ± 0.34 Hz, Laser: 1.98 ± 0.29 Hz, Monochrome: 1.83 ± 0.26 Hz; Fig. 5b). The results indicated that the frequency of periodic eye movements was not decreased in the laser and monochrome conditions compared to the control condition.

The SD of head tilt angle showed a marginally significant difference across conditions (*F*(1.364, 19.093) = 3.903, *p* =.052, *η*_*p*_*²* = 0.536). Post hoc comparisons indicated a marginally significant reduction in SD of head tilt angle in the laser condition compared to the monochrome condition (*p* =.072) (Control: 3.18 ± 0.52 deg, Laser: 2.37 ± 0.21 deg, Monochrome: 3.49 ± 0.45 deg).

**Fig. 6:**
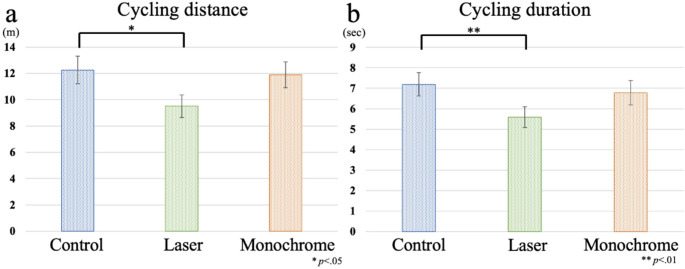
**a** Cycling distance and **b** cycling duration in the control, laser and monochrome conditions. The error bars represent the standard error (SE)

Cycling speed was not significantly different among conditions (*F*(1.452, 20.333) = 0.344, *p* =.645, *η*_*p*_^*2*^ = 0.024) (Control: 1.72 ± 0.1 m/s, Laser: 1.70 ± 0.09 m/s, Monochrome: 1.75 ± 0.09 m/s). In contrast, cycling distance was significantly different among conditions (*F*(2, 28) = 5.470, *p* =.01, *η*_*p*_^*2*^ = 0.281; Fig. 6a). Post hoc comparisons showed that cycling distance in the laser condition was significantly shorter than that of control condition (*p* =.02) (Control: 12.26 ± 4.11 m, Laser: 9.51 ± 3.30 m, Monochrome: 11.89 ± 3.79 m). Similarly, cycling duration showed a significant main effect of condition (*F*(2, 28) = 6.688, *p* =.004, *η*_*p*_^*2*^ = 0.323; Fig. 6b). Post hoc comparisons revealed that cycling duration in the laser condition was significantly shorter than in the control condition (*p* =.004) (Control: 7.19 ± 2.20 s, Laser: 5.58 ± 1.96 s, Monochrome: 6.78 ± 2.32 s).

## Discussion

This study aimed to investigate the impact of the visual environments that manipulate gaze behavior on cyclists’ dynamic balance control. Therefore, we employed a laser fixation point and a monochromatic path to assess changes in eye movements and cycling performance. The results showed that the amplitude at the peak frequency of eye movements was significantly lower in the laser condition than in the control condition. Additionally, both cycling distance and cycling duration were shorter in the laser condition compared to the control condition. These results suggest that fixation on a laser point projected onto the path suppresses the eye movement amplitude and impairs balance control during cycling.

The amplitude at the peak frequency of eye movements was significantly lower in the laser condition than in both the control and monochrome conditions. This result indicated that the amplitude of eye movements was suppressed under the laser condition, as we hypothesized. During locomotion, it was reported that using a heads-up display to present a fixation point in the forward field of view suppressed eye movement (Moore et al. 2008). It was also known that fixating on a point suppressed the amplitude of OKR in experiments using visual stimuli (Pola et al. 1992). In our study, there was no difference in the peak frequency of eye movement, which appeared to be consistent with previous studies indicating that fixation at a point did not affect the frequency of OKR (Pola et al. 1992). Although fixation on laser did not alter the peak frequency of eye movements, it led to a reduction in their amplitude, suggesting a diminished stabilizing function of OKN on the visual environment. Furthermore, the SD of head tilt angle showed no difference among the conditions. The mean values were 3.18 deg in the control condition and 2.37 deg in the laser condition, both of which are close to the value reported in a previous study using straight and narrow path cycling (2.51 ± 0.81 deg; Kojima and Kokubu 2024), indicating that head movement was also small in the present study. These findings suggest that fixation on the laser suppressed eye movements rather than head movements, thereby altering the visual environment. In summary, the visual environment designed to manipulate gaze behavior using a fixation point projected on the path at a fixed distance suppressed the amplitude of OKN, and this may have altered the velocity vector of retinal flow.

The cycling distance and duration in the laser condition were significantly shorter than those in the control condition. These results suggest the possibility that dynamic balance during cycling was deteriorated by fixation, as hypothesized. Previous studies have reported that compensatory eye movements, such as OKR, are predominantly observed during cycling on a straight and narrow path, whereas travel fixation, in which eye movements are suppressed, is rarely observed (Vansteenkiste et al. 2013; Kojima and Kokubu 2024). Compensatory eye movement contributes to the suppression of retinal slip and the stabilization of the fovea (Lappi 2016; Matthis et al. 2022; Muller et al. 2023). Suppression of these eye movements may disrupt visual information and negatively affect motor control. Interestingly, our results differed from those of previous studies on static balance control. In tasks evaluating static balance, fixation on a point in front has been shown to reduce body sway (Guerraz et al. 2000; Laurens et al. 2010). For example, fixation on a distant point has also been reported to reduce both the distance and velocity of the center of pressure (Lee and Lishman 1975). This stabilizing effect of fixation is attributed to the contribution of extraocular muscle signals associated with eye movements (Jahn et al. 2002; Strupp et al. 2003; Glasauer 2005). These different results may be attributed to the position of the fixation point and the presence or absence of movement in the participants. In our study, the fixation point was set on the ground, and the participants rode a bicycle and controlled their balance using their entire body. Additionally, cycling speed was not significantly different among conditions. The mean cycling speed observed in all conditions was approximately 1.72 m/s (range: 0.96–2.37 m/s), which is below the 4.3 m/s required to maintain independent stability (Meijaard et al. 2007). This indicates that the cyclists were required to maintain dynamic balance during cycling. During self-motion, retinal flow arises from a combination of optic flow generated by movement through the environment and flow resulting from eye movements. Our findings partially support the idea that compensatory eye movements, which stabilize the visual field, are necessary for dynamic balance control that relies on complex retinal flow during more dynamic self-motion.

An alternative explanation for the observed decrease in cycling stability due to fixation can be discussed from the perspective of attentional demands. The cycling task employed in this study was relatively challenging, as reflected by the mean cycling distance of 12.26 ± 4.11 m in the control condition. Although cycling is often considered an automated motor skill, it is likely that the task imposed a considerable cognitive load on participants. According to the attentional capacity model (Kahneman 1973), allocating attentional resources to maintain fixation on a specific point may have interfered with the attention necessary to effectively perform the cycling task. This notion is supported by evidence from walking studies, where even automated locomotion requires attentional resources, and dual-task interference negatively affects walking performance, especially in older adults with impaired balance (Woollacott and Shumway-Cook 2002; Beurskens and Bock 2012). Furthermore, the fixation point was fixed at a constant distance ahead, which may also have caused the observed destabilization during cycling. Instructing participants to fixate on the laser point likely caused their attention to be narrowly focused on a nearby location. In contrast, it is well documented that directing attention toward an external, more distant point can enhance balance control (Park et al. 2015), with improvements increasing as the attentional focus distance grows (McNevin et al. 2003). Therefore, in the laser fixation condition, attentional focus may have been constrained to a nearer distance compared to the control condition, possibly resulting in decreased cycling stability.

We also attempted to suppress eye movements without constraining the gaze position by using a monochrome cycling path. However, the amplitude at the peak frequency of eye movements and the peak frequency did not differ between the control and monochrome conditions. The results suggest that the monochrome path did not suppress eye movements. The reason why eye movements occurred even under monochrome conditions might be the outdoor experimental setting; this was probably due to the lack of control over the texture of the surrounding ground. It has been reported that eye movements are not only triggered by objects captured in the central retina but also by the stimulus in the peripheral retina, leading to a high OKN gain at stimulus velocities under 30 deg/s (Howard and Ohmi 1984). In our study, since cycling was performed at low speeds, stimuli from the peripheral retina may have contributed to the induction of OKN. Therefore, the visual environment designed to manipulate the gaze using the narrow path of a monochromatic surface did not suppress OKN induction. The fact that the amplitude of OKN was not suppressed under the monochrome condition means that we could not completely rule out the possibility that the decrease in cycling distance and duration under the laser condition was due to fixation of the gaze position. The fixation distance used in the laser condition in this study was based on the average value from a previous study on cycling through a straight narrow path (Kojima and Kokubu 2024). In the previous study, the gaze angle was − 22.2 deg, and the eye height during cycling was 1.60 m, suggesting that the participants were focusing on a path approximately 3.92 m ahead. Therefore, in this study, we set the fixation point at a fixed distance of 4 m ahead for all participants. This approach was intended to minimize the possibility that constraining the gaze position would reduce the stability of cycling. However, future experiments are needed to clearly distinguish the effects of eye movements suppression and gaze position.

This task was deliberately chosen to model real-world situations that place heightened demands on balance control such as navigating poorly maintained roads, wet or slippery surfaces, or riding close to vehicular traffic. Under these physically challenging conditions, cyclists tend to direct their gaze more toward the road surface (Vansteenkiste et al. 2013, 2014). For instance, Vansteenkiste et al. (2014) found that cyclists riding on unpaved paths spent approximately 63% of the time looking at the ground, compared to only 8% looking straight ahead. This gaze behavior is consistent with our task setup and supports its ecological relevance. Our findings have important practical applications. For example, looking at a smartphone screen while cycling, walking, or riding a motorcycle can increase lateral deviation and pose a safety risk. It would be wise to avoid looking at electronic device screens, especially for navigation, in situations with crowded, narrow, or unpaved road conditions. Our findings suggest natural gaze behavior is likely to be an important factor for cycling safely.

The present study has three limitations. First, it remains unclear whether the suppression of eye movements directly caused the decrease in cycling stability. To investigate this potential causal relationship, future studies could involve participants cycling under natural viewing conditions, while simultaneously recording gaze data and cycling stability indicators such as lateral deviation, steering variability, and trajectory. By conducting time-series analyses to determine whether travel fixations　(i.e., gaze behaviors characterized by suppressed eye movements) precede instances of instability, it may be possible to infer a causal link between eye movement suppression and decreased balance control. Second, the study lacks a monochrome condition using a laser. In this experiment, the number of conditions was limited to three in order to prevent participants’ fatigue. This comparison would help clarify whether the differences in head tilt angle observed between the laser and monochrome conditions are primarily driven by gaze fixation or by the visual texture of the path. Third, not only eye movements but also gaze position were manipulated in the laser condition. Thus, the manipulated gaze position may have impaired balance control. In this experiment, the gaze position of the laser was determined based on previous studies about cycling on a straight and narrow path. Future research should investigate the impact of different gaze positions, such as 2–6 m ahead, on balance control.

## Conclusion

This study examined whether two types of visual environments that manipulate gaze could suppress eye movements and impair cyclist’s dynamic balance control. The results showed that the amplitude at the peak frequency under the laser condition was lower than those under the control and monochrome conditions. The cycling distance and duration under the laser condition were shorter than those under the control condition. However, there were no significant differences in eye movements or cycling performance between the control and monochrome conditions. These results suggest that fixation on a laser projected onto a path suppresses the amplitude of eye movements and impairs balance control in cycling. Further research is needed to clarify the extent to which these compensatory eye movements contribute to dynamic balance control.

## Data Availability

The data and materials related to this study are available at the following OSF repository: https://osf.io/jtb9n/.
